# Influence of Granular Activated Carbon Addition on Methane Production in Dry and Semidry Anaerobic Digestion of Swine Manure

**DOI:** 10.1021/acsomega.5c02516

**Published:** 2025-07-07

**Authors:** Amanda de Sousa e Silva, Amanda Lima Moraes dos Santos, Isabele Clara Cavalcante Malveira, Bianca Holanda Albano Girão, André Bezerra dos Santos

**Affiliations:** Department of Hydraulic and Environmental Engineering, Federal University of Ceará, Campus do Pici, Bloco 713, CEP:, Fortaleza, Ceará 60455-900, Brazil

## Abstract

This work investigated the effect of granular activated carbon (GAC) on methane-rich biogas production in semidry (10% total solids, TS) and dry (15% TS) anaerobic digestion (AD) of swine manure. The conductive material GAC can improve methane production exponentially in AD, even without pretreatment. However, in semidry and dry conditions, there was no significant difference in methane production yield when using raw or pretreated swine manure with the addition of 20 g/L GAC. For this condition, when using the pretreated substrate, the main observed effect was related to the lag phase increase from 6.5 to 10.6 days and from 10.4 to 32.0 days at 10 and 15% TS, respectively. In semidry AD, with pretreated manure, 10 g/L GAC addition yielded 190 mL CH_4_/gVS, a value 21% higher than with 20 and 30 g/L GAC. In dry AD, the best condition was with 30 g/L GAC (157 mL CH_4_/gVS), followed by 20 and 10 g/L GAC (127 and 18 mL CH_4_/gVS, respectively). Therefore, the TS content influences the ideal GAC concentration. About microbiology, the increase in the abundance of phyla *Acidobacteriota*, *Bacteroidota*, *Chloroflexi*, and *Firmicutes* was linked to greater methane production when using raw manure. The dominant genus of bacteria in most reactors was *Georgenia*, and the main abundances of archaea were *Methanobacterium* (slightly more favored with GAC addition) and *Methanosaeta*. Therefore, GAC increases methane production not only by favoring certain important species in AD but also mainly by promoting Direct Interspecies Electron Transfer (DIET) and adsorbing inhibitory compounds, thereby improving methane-rich biogas production.

## Graphical abstract

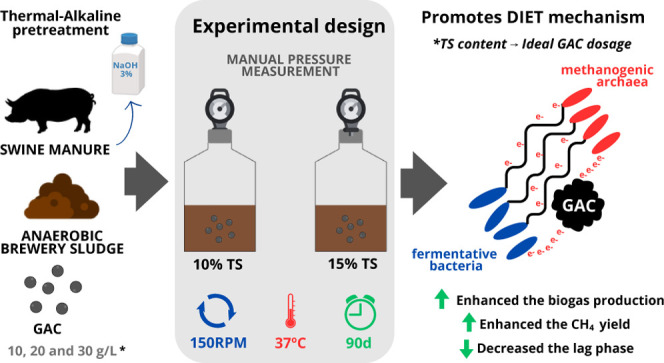

## 1 Introduction

According to the United States Department of Agriculture, pig farming is one of the world’s largest agricultural activities, with global animal protein exports totaling 12.3 million tons in 2022.^1^ It is estimated that approximately 300 million liters of liquid pig manure are generated daily, containing a variety of pollutants, including organic matter, nitrogen, phosphorus, heavy metals, antimicrobials, and pathogens.^2,3^ Therefore, if not managed properly, such pollutants can contaminate the soil, groundwater, and surface water, besides contributing to air pollution by releasing ammonia and other gases. Then, correctly managing organic waste is a major challenge for the livestock sector.

Swine manure, abundant in proteins, lipids, and carbohydrates, serves as an optimal substrate for biogas production through anaerobic digestion (AD), a biotechnological process capable of converting organic matter present in manure into biogas rich in methane, which can be converted into heat and electricity, reducing dependence on fossil fuels and emissions associated with its use.^4^ The AD could be classified based on the total solids (TS) content in wet (TS < 10%), semidry (10% ≤ TS < 15%), or dry (TS ≥ 15%). This TS content influences the type of reactor to be used, energy consumption, digestate final quality, the different endogenous inhibitions during biodegradation, and process profitability.^5,6^ Dry and semidry AD have advantages over wet AD, such as the possibility of execution in smaller reactors, reduced water use, and energy savings for heating.^7^ However, it presents biological and technological disadvantages due to the excess of solids, such as the difficulty of mixing and homogenizing the medium, which consequently affects methane production due to the deficiency in the diffusive transport of soluble and intermediate compounds.^8^

AD comprises four stages: hydrolysis, acidogenesis, acetogenesis, and methanogenesis, in which bacteria carry out the first three steps, while archaea do the last. The syntrophy between the microorganisms involved in the AD process must be improved. Fermentative bacteria and methanogenic archaea have complementary metabolisms, requiring syntrophy for electron exchange, which can be indirect or direct.^9^ The indirect pathway that transfers electrons through diffusive transport from chemical compounds (hydrogen or formate) is considered a bottleneck for producing CH_4_ since it occurs only when there is low partial pressure of H_2_, a phenomenon regulated by hydrogenotrophic archaea. It is a thermodynamically unfavorable pathway for methane generation, leading to volatile fatty acids (VFAs) accumulation and thus reducing AD efficiency.^10,11^ In contrast, the direct pathway—Direct Interspecies Electron Transfer (DIET)—is considerably faster and more energy-efficient than indirect mechanisms as it does not require complex enzymatic steps to produce, consume, and diffuse redox mediators.^12^ This direct mechanism can occur through biotic (cytochromes and conductive pili) and abiotic (mediated by conductive materials) pathways.^10^

So, a promising technique to optimize anaerobic digestion is the addition of conductive materials, which can be metal-based, such as iron oxide and hematite, or carbon-based, such as granular activated carbon (GAC) and biochar.^13^ The last ones, due to their larger size in relation to the microorganisms involved in the process, allow the connection of multiple microorganisms and enable interaction without physical contact between the bacteria that donate electrons and the archaea that receive them.^14^ Thus, the connection with the conductive material is sufficient to promote the DIET.^15^ Additionally, GAC is a low-cost, lightweight, chemically stable conductive material with high biocompatibility, which can act as an adsorbent for toxic compounds and allow the fixation of microorganisms without forming aggregates.^16,17^ Furthermore, GAC supplementation can increase the abundance of genes relevant to DIET (e.g., pilA and omcS).^18^ However, the effects of adding GAC and the optimal concentration must be studied as a high concentration of GAC adsorbs COD substances and reduces the substrates available for methane conversion.^19^

Additionally, for a greater understanding of this entire biochemical process, kinetic analysis based on mathematical models is an important tool for predicting methane production, designing, and optimizing reactor performance.^3^ The result of this analysis enables the quantification of the impact of variables and inhibitions that occurred during the bioprocess on the biogas production rate and yield.^20^

Studies have shown that adding GAC enhances the efficiency of dry AD of swine manure, increasing the biogas production rate.^21,22^ However, this process still needs to be better understood and the ideal dosage of GAC requires clarification. In addition, the combined evaluation of the pretreatment of swine manure and the addition of GAC to the process was not found in the literature. Various pretreatment strategies—ranging from physical and mechanical to thermal, chemical, or biological approaches—are employed to reduce substrate particle size, enhance surface area availability, alter its physicochemical characteristics, and improve substrate accessibility to microbial consortia. A thermoalkaline pretreatment is interesting because it combines the advantages of thermal and alkaline processes, allowing faster reactions using lower temperatures and lower doses of alkalizing agents.^23^ Various alkaline chemicals have been used for pretreatment of lignocellulosic feedstocks, with NaOH frequently identified as the most cost-effective.^24–27^

Therefore, this work aimed to evaluate strategies for enhancing the anaerobic biometalation of swine manure in anaerobic digestion. For this, the effect of SM thermoalkaline pretreatment and the addition of granular activated carbon in different dosages on the dry (15% TS) and semidry (10% TS) AD of swine manure was investigated. Kinetic modeling analysis was employed to gain a deeper understanding of the influence of the evaluated parameters and to derive important kinetic coefficients for optimization and scale-up. Additionally, molecular biology techniques were employed to gain a deeper understanding of the diverse microbial populations and the dynamics of the ecological changes.

## 2 Materials and Methods

### 2.1 Substrate and Inoculum

The substrate (SM) was collected in a pig farm located at the Federal University of Ceará (Fortaleza, CE), in the Department of Animal Science, by scraping the residue in the bays.

Soon after collection, this raw manure underwent physicochemical characterization through analyses of solids (total solids, TS, and volatile solids, VS), pH, chemical oxygen demand, and total ammonia nitrogen of the soluble fraction (COD_S_ and TAN_S_) (Table S1) and was then refrigerated at 4 °C until use. Swine manure underwent thermal-alkaline pretreatment using 3% w/v NaOH at a ratio of 60% mass/volume, followed by incubation in an autoclave at 121 °C for 30 min. The anaerobic inoculum was sludge collected from the wastewater treatment plant of a brewery located in Pacatuba, Ceará, Brazil. The physical–chemical characterization of the inoculum consisted of the same parameters used for SM. Before the solids analysis, the sludge was dewatered through sieves to obtain a higher total solids content (Table S1).

### 2.2 Conductive Material

Commercial granular activated carbon (12–20 mesh) (Sigma-Aldrich, St. Louis, MO, USA) was used as conductive material. The particle size was 0.8–1.7 mm, the bulk density was 1.8–2.4 g/cm^3^, and the surface area was 650 m^2^/g. The GAC was washed three times with deionized water to remove impurities and dried in an oven at 105 °C for 24 h to remove moisture. In order to use a material without electrical conductivity for comparison, granular nylon of size 1.1–2.4 mm and apparent density of 1.1 g/cm^3^ was used.

The chemical composition of granular activated carbon was analyzed using X-ray Fluorescence (XRF) with a Rigaku wave-dispersive sequential X-ray spectrometer (model ZSX mini II), according to Alexandre et al. (2024). Structural characterization of the isolated and synthesized products was carried out via Fourier Transform Infrared Spectroscopy (FTIR) using an IR-Prestige-21 SHIMADZU spectrometer. FTIR spectra were recorded from KBr pellets across the spectral range of 400–4000 cm^–1^ with a resolution of 4 cm^–1^ in absorbance mode.

The zeta potential of the GAC was determined by using a Zetasizer Nano ZS (Malvern Instruments Ltd.). Before the measurements, the sample was stabilized for 120 s. The analysis included five independent runs, each comprising 10–100 recordings. Data collection was halted when the instrument detected repetitive and consistent results, thus ensuring accuracy based on the preset conditions of the device.

Surface morphology was examined by using scanning electron microscopy (SEM) combined with energy-dispersive X-ray spectroscopy (EDS) on an Inspect S50 microscope (FEI Company, USA). Samples were mounted on aluminum stubs with double-sided carbon tape and coated with a thin gold layer under low-pressure argon by using Quorum equipment (model Q15DT ES).

### 2.3 Biochemical Methane Potential Tests

The experiments were conducted in batch mode, with triplicate samples, in borosilicate bottles with a working volume of 70 mL. The concentrations of TS were 10% and 15% for the semidry and dry AD, respectively, adjusted with the addition of deionized water. All assays were prepared with a substrate/inoculum ratio of 1 gVS_substrate_/gVS_inoculum_, macro- and micronutrient solution addition according to Angelidaki et al. (2009), pH adjustment to approximately 7 if necessary, and addition of sodium bicarbonate (0.1 g/gTS) to buffer the system.

The control reactors were made up of sludge at 10% TS (CT_S_); sludge and glucose (0.35 gCOD/gVS) at 10% TS (CT_SG_); and sludge, glucose (0.35 gCOD/gVS), and GAC −20 g/L – at 10% TS (CT_SG‑GAC_). The other media contained sludge and raw SM at 10 and 15% TS without additive (10RSM and 15RSM) and with 20 g/L GAC (10RSM_GAC20_ and 15RSM_GAC20_); and sludge and pretreated SM at 10 and 15% TS without additive (10PSM and 15PSM), with nylon (10PSM_N_), and with 10 g/L GAC (10PSM_GAC10_ and 15PSM_GAC10_), 20 g/L GAC (10PSM_GAC20_ and 15PSM_GAC20_), and 30 g/L GAC (10PSM_GAC30_ and 15PSM_GAC30_). In Table S2, the amounts of inoculum, substrate, and additive used in each condition are presented.

The bottles were sealed with butyl rubber stoppers and purged with nitrogen (N_2_) for 1 min to establish an anaerobic environment. They were then placed in a shaker incubator (MA-420, Marconi LTDA, Brazil) and agitated at 150 rpm under mesophilic conditions (37 °C) for 90 days until methane production reached a stable state.

### 2.4 Analytical Methods

pH and solids series of the raw samples were analyzed at the experiment’s beginning and end. The samples were mixed with deionized water in a proportion of 1:4 and then centrifuged at 13,000 rpm for 10 min (Eppendorf AG, Germany) followed by filtration through a glass fiber membrane with a pore size of 0.45 μm (EMD Millipore, USA) to proceed with the analyses of COD_S_, TAN_S_, and Total Organic Carbon—TOC_S_ (TOC-L CSN, Shimadzu Corporation, Japan).

Biogas quantification was carried out by measuring the gauge pressure in each reactor. The biogas composition was evaluated by using gas chromatography with dielectric barrier ionization discharge (GC BID-2010 Plus, Shimadzu Corporation, Japan), as described by Morais et al. (2020).

### 2.5 Kinetic Modeling and Statistical Analysis

Several kinetic models—First and Second order, Modified Gompertz, Monomolecular, Transfer, and Logistic—were used to describe the anaerobic digestion bioprocess of swine manure.^28^ Parameters for these models were estimated using nonlinear least-squares analysis conducted with the Solver tool in Microsoft Excel 2021. The model that best fits the bioprocess was selected based on the coefficient of determination (*R* ^2^) and Akaike Information Criterion (AIC).^29^

All work results were statistically analyzed using Origin 8.1 software (Microcal Software Inc., Northampton, MA, USA). Analysis of variance (ANOVA) was performed with a 95% confidence level and a 5% significance level (*p* < 0.05). Tukey tests were used to compare the different GAC dosages (10, 20, and 30 g/L). The data were exhibited as average values, followed by a letter indicating the statistical treatment; identical letters denote no significant difference at *p* < 0.05.

### 2.6 Microbial Community Analysis

Genetic sequencing and data processing, along with bioinformatics analysis, were conducted following the methods described by Silva et al.^23^ The raw sequence data generated in this study have been submitted in the National Center for Biotechnology Information (NCBI) BioProject database under ID PRJNA1090783 (https://www.ncbi.nlm.nih.gov/bioproject/1090783).

## 3 Results and Discussion

### 3.1 Conductive Material Characterization

FTIR analysis of granular activated carbon (GAC) revealed a diversity of chemical functional groups (Figure 1). The intense band at 3417 cm^–1^ indicates the presence of hydroxyl (−OH) groups, which are known to enhance the adsorption of polar compounds. The peak at 1566 cm^–1^ corresponds to C=C bonds, which are typical of the aromatic structures commonly found in carbonized materials.^30–34^ The peak at 1107 cm^–1^, associated with the C–O–C functional group, was particularly prominent, suggesting an abundance of oxygenated functionalities. Additionally, the peak at 806 cm^–1^, attributed to out-of-plane C–H bending vibrations in aromatic rings, indicated the presence of polycyclic or condensed aromatic structures.^35–37^ Oxygen functionalization enables specific chemical interactions, including hydrogen bonding and dipole–dipole interactions, rendering the material highly versatile for environmental applications.

**Figure 1:**
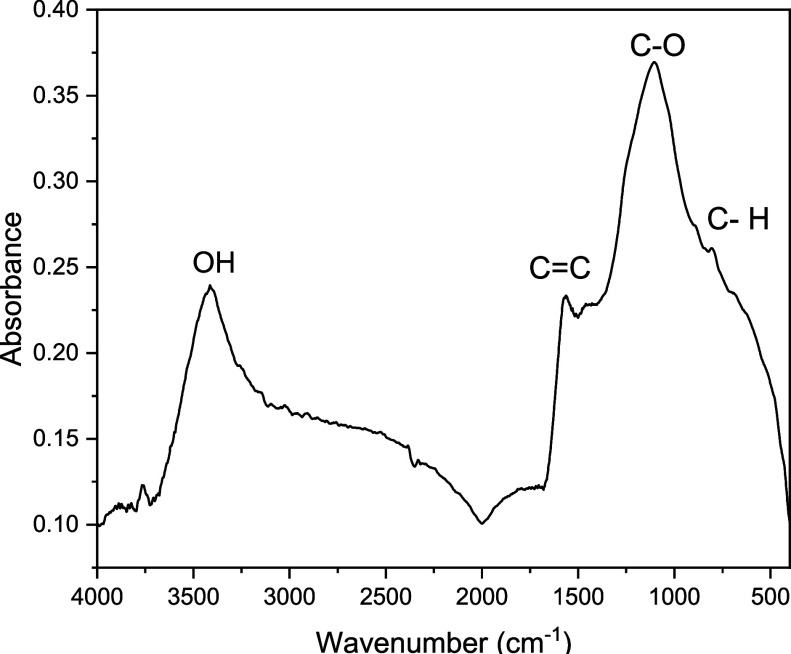
FTIR spectra of granular activated carbon.

SEM images of GAC (Figure 2) reveal an amorphous surface morphology with a heterogeneous distribution of macropores and mesopores as well as a rough texture typical of activated carbon materials. Although micropores (<2 nm) cannot be directly observed by SEM, their presence is inferred from the high specific surface area (650 m^2^/g) reported by the manufacturer and supported by prior studies on similar commercial GAC.^38^ This high porosity is promoted by the activation process, which is essential for the adsorption of compounds in environmental applications. These observations are consistent with previous studies, which highlight that materials with high microporosity and mesoporosity provide an ideal combination of adsorption capacity and accessibility to internal surfaces.^37,39^

**Figure 2:**
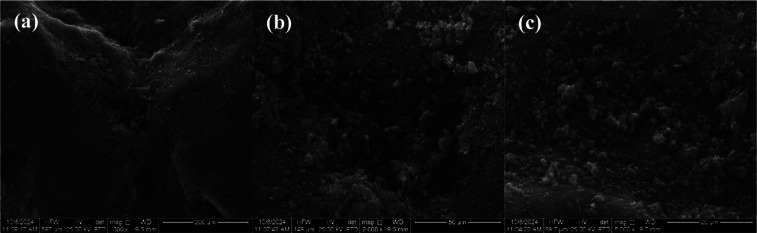
SEM images of granular activated carbon at different magnification levels: (a) 500×, (b) 2000×, and (c) 5000×.

XRF analysis (Table S3) revealed a significant presence of TiO_2_ (12.3%), Al_2_O_3_ (7.64%), and CaO (11.83%). The notable presence of TiO_2_ is particularly relevant as recent studies suggest that it can enhance the adsorption of polar compounds because of its semiconducting properties and ability to interact with oxygenated functional groups.^39^ Additionally, the high SiO_2_ content (35.66%) contributed to the mechanical stability and adsorption capacity of the nonpolar organic compounds.

Compared with previous studies, activated carbons derived from biomass with high levels of oxygenated functional groups and high porosity are effective in removing contaminants such as heavy metals and persistent organic compounds. For instance, Bahsaine et al. (2024) reported that carbons with significant proportions of C–O–C and–OH exhibit superior performance in adsorbing polar pollutants. Similarly, Gwenzi et al.^37^ emphasized that the presence of TiO_2_ contributes to photocatalytic applications, enhancing the versatility of activated carbon.

The zeta potential values for activated carbon ranged from −19 to −22.3 mV, indicating a highly negative surface charge. This behavior is attributed to the deprotonation of acidic functional groups, such as carboxyls, which increases the electrostatic repulsion between particles, thereby enhancing the colloidal stability.^40,41^ A negative zeta potential enhances electrostatic repulsion, prevents particle aggregation, and ensures better dispersion of activated carbon in aqueous media.^42,43^ These results highlight the importance of ionic strength regulation in optimizing the functionality of activated carbon in bioprocesses.^44^ A high negative charge enhances microbial retention and promotes biofilm formation, which is fundamental for the efficiency of anaerobic AD.^41,43^

In AD, the colloidal stability of activated carbon plays an important role in the microbial activity. Studies have shown that negatively charged materials can enhance the retention and activity of microbial consortia, particularly methanogenic archaea, which are essential for biogas production.^41,43^ The high surface charge and dispersion capacity of activated carbon can facilitate microbial adhesion, provide a stable biofilm environment, and reduce the washout of microbial populations in continuous anaerobic systems.

Additionally, the high negative charge of GAC in anaerobic digestion systems may contribute to buffering capacity and interspecies electron transfer (IET), particularly through DIET. This mechanism plays a key role in enhancing methane production by facilitating more efficient interactions between fermentative bacteria and methanogens.^40^ DIET has been recognized as a major pathway for improving methane production by reducing the reliance on mediated electron transfer via hydrogen or formate, resulting in faster reaction kinetics in AD.^44^ Nevertheless, additional modifications, such as surface functionalization or optimization of operating conditions, may be required to maximize the positive effects of activated carbon in AD applications.^41^ This result suggests that GAC can promote colloidal stability and enhance its potential application in AD. This property can improve microbial retention, electron transfer, and process stability, ultimately optimizing the biogas production efficiency.

### 3.2 Organic Matter Degradation and Methane/Biogas Yield

Table 1 presents the TS, VS, and TOCs data at the beginning and end of the experiment for each condition evaluated. There was a greater reduction in total and volatile solids in reactors with GAC addition in both semidry and dry AD. However, this reduction was greater in semidry conditions, indicating greater organic matter removal. Concerning the total organic carbon of the soluble fraction (TOCs), in control reactors with glucose, the final concentration of TOCs decreased compared to the initial concentration, which can be explained by the consumption of this soluble substrate throughout the bioprocess.

**Table 1:** Analysis of the Solid Content and Total Organic Carbon of the Soluble Fraction

reactors	TS (%)		VS (%)		TOCs (mg/L)	
	initial	final	initial	final	initial	final
CT_S_	12.4	11.2	9.4	8.4	771	1195
CT_SG_	11.8	9.6	9.3	6.7	6516	932
CT_SG‑GAC_	13.4	11.6	9.6	8.8	5638	1066
10RSM	11.1	9.5	6.6	6.8	5328	11,687
10RSM_GAC20_	12.2	9.0	8.7	6.2	4684	1664
10PSM	10.9	9.7	6.5	6.5	7356	12,743
10PSM_N_	11.3	10.3	6.9	7.2	6410	12,612
10PSM_GAC10_	11.5	8.4	7.1	5.2	7460	4405
10PSM_GAC20_	12.5	8.7	8.6	5.4	6744	2695
10PSM_GAC30_	13.4	9.9	8.8	7.0	6388	2168
15RSM	15.2	14.1	11.3	10.7	5990	15,284
15RSM_GAC20_	17.3	15.2	13.1	11.5	5813	4047
15PSM	14.5	13.2	8.4	9.5	9195	15,327
15PSM_GAC10_	15.3	13.4	11.1	9.8	6994	15,493
15PSM_GAC20_	16.1	12.7	11.5	8.3	6323	6393
15PSM_GAC30_	17.1	12.7	11.1	9.0	5989	4182

Conversely, there was no difference when adding GAC, which corroborates the similar amounts of methane produced in CT_SG_ and CT_SG‑GAC_ (Table 2). However, in Figure 3A, it is possible to observe that GAC promoted a difference in the methane production curve, making it more pronounced in the first days of the experiment and providing a shorter lag phase. Although the maximum daily methane yield was reached by CT_SG_ on the first day (5.8 mL of CH_4_/gVS·d), a new peak was observed again on the 21st day (4.3 mL of CH_4_/gVS·d). Conversely, CT_SG‑GAC_ showed an increasing trend in daily methane yield until the 10th day, when it reached its maximum value (7.0 mL of CH_4_/gVS·d). Therefore, the condition with GAC showed higher daily methane production until the 12th day and had a methane production curve with a greater slope of the straight line.

**Table 2:** Biogas and Methane Yields and Methane Content in Biogas

reactors	biogas yield (mL/gVS)	methane yield (mL/gVS)	methane content (%)
CT_S_	111^a^	69^a^	62^a^
CT_SG_	189^b^	96^b^	51^b^
CT_SG‑GAC_	185^b^	95^b^	51^b^
10RSM	69^a^	3^a^	5^a^
10RSM_GAC20_	276^b^	154^b^	56^b^
10PSM	73^a^	3^a^	4^a^
10PSM_N_	76^a^	3^a^	4^a^
10PSM_GAC10_	361^c^	190^c^	53^b^
10PSM_GAC20_	276^b^	155^b^	56^b^
10PSM_GAC30_	276^b^	158^b^	57^b^
15RSM	56^a^	2^a^	4^a^
15RSM_GAC20_	201^d^	111^d^	55^b^
15PSM	97^a^	8^a^	8^a^
15PSM_GAC10_	72^a^	18^a^	25^c^
15PSM_GAC20_	251^b,d^	127^d^	51^b^
15PSM_GAC30_	290^b^	157^b^	54^b^

**Figure 3:**
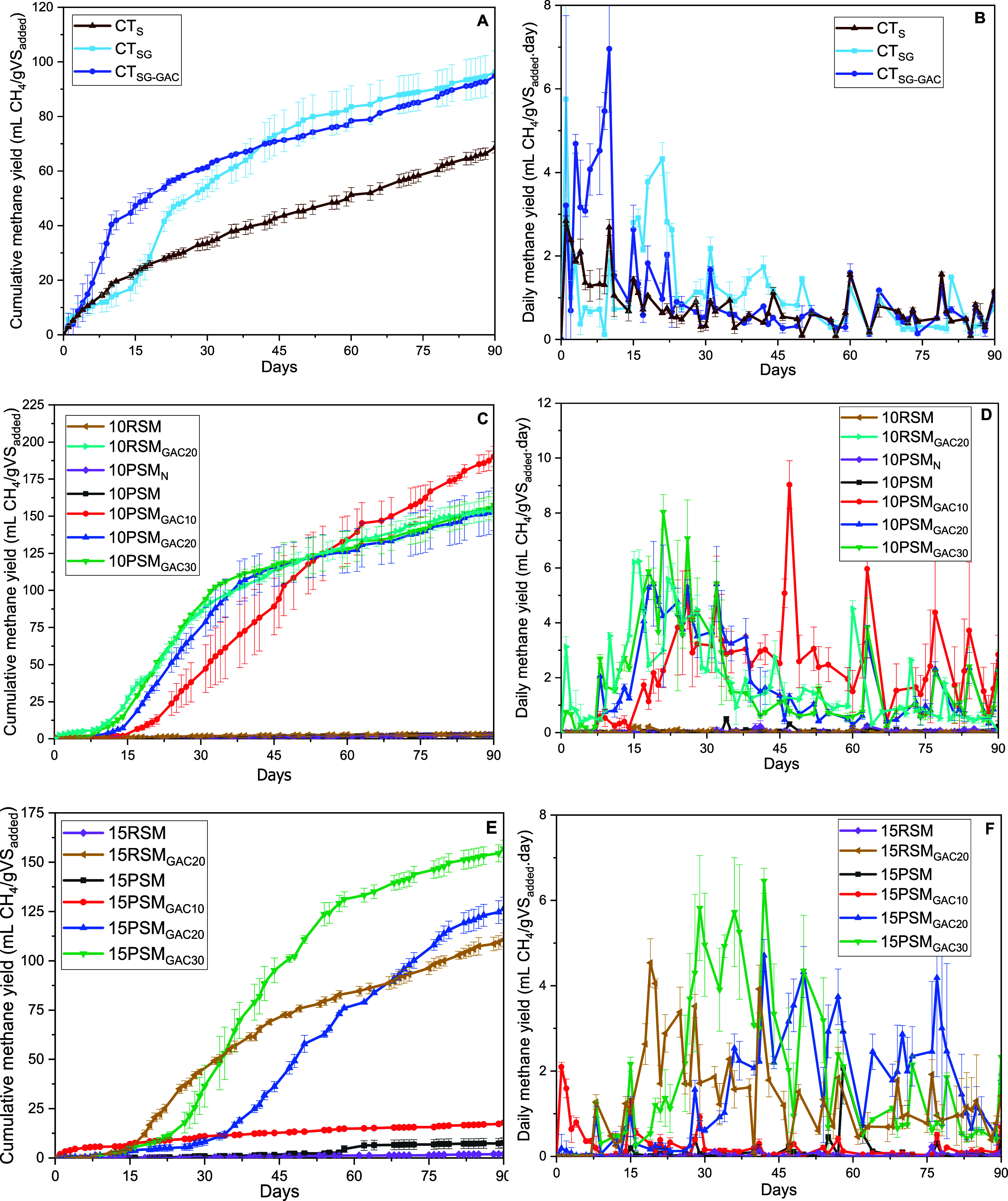
Cumulative and daily methane yield of reactors of sludge control (A,B), semidry (C,D), and dry (E,F) anaerobic digestion.

In semidry AD, the reactors with pretreated manure without additives (10PSM) and with nylon (10PSM_N_) showed an increase of 67–93% in TOCs (soluble organic matter), while, for the reactors with GAC (10PSM_GAC10_, 10PSM_GAC20_, and 10PSM_GAC30_), there was a 42–66% reduction of TOCs. The TOC increase phenomenon is explained by the initial hydrolysis of organic matter, which occurred in all reactors. However, its reduction occurs through the conversion of organic matter into methane, which is effective only in reactors supplemented with GAC.

The control group 10PSM_N_ with nylon, a nonconductive material, intended to verify whether the GAC effect was due to its capacity as a conductive material or just because it is a support medium that allows biofilm formation. The biogas and methane production yield of the nylon condition (10PSM_N_–76 and 3 mL/VS of biogas and CH_4_) was like that of the condition without additive (10PSM–73 and 3 mL/VS of biogas and CH_4_). When adding 20 g/L GAC (10PSM_GAC20_), the biogas and methane yields increased to 276 and 155 mL/VS, respectively, representing a 2.8-fold increase in the accumulated biogas yield and an exponential increase in methane production.

This result demonstrates that even when added at the same concentration, the conductive characteristics of GAC prevailed over the physical support material property also present in nylon, reinforcing the role of conductivity in DIET. Unlike inert materials, conductive carbon-based materials such as GAC allow the connection of multiple microorganisms and enable electron exchange without the need for direct physical contact between electron-donating bacteria and electron-accepting archaea. This feature enhances syntrophic interactions and accelerates the methanogenesis process by promoting a more efficient interspecies electron flow. In addition, the adsorption capacity of GAC, elucidated in the previous topic of conductive material characterization, contributes greatly to the increase in methane production by adsorbing compounds that inhibit the methanogenesis process, as will be discussed in the next section.

The favoring of methanogenesis in the presence of GAC is also linked to the promotion of direct interspecies electron transfer and other characteristics of GAC that can significantly contribute to increased methane production.^13^ Carbon-based materials can also adsorb hydrogen generated by oxidizing bacteria during AD, thereby decreasing the partial pressure of hydrogen within the biodigester.^45^ This capability helps mitigate potential imbalances in the AD system caused by excessive hydrogen accumulation.

Additionally, the porous structure of GAC, with a high level of porosity promoted by the activation process as discussed previously, plays an important role because it facilitates the formation of biofilms on its surface, promoting syntrophy between microorganisms and contributing to changes in the microbial community.^38^ For example, the enhancement of the acetoclastic pathway through the addition of GAC is a highly important finding, considering that this pathway may account for more than 90% of methane production in anaerobic digestion.^19,46^ Furthermore, GAC can also increase the presence of electroactive bacteria, boosting methane production in AD.^47^

Conductive materials such as GAC contain trace elements (iron, manganese, calcium, magnesium, and potassium), as can be observed by XRF analysis (Table S3), which play a critical role in the formation and function of key enzymes involved in microbial metabolism. By supplying essential nutrients, GAC can stimulate microbial growth, activity, and cometabolism, ultimately enhancing key processes such as hydrolysis, acidification, and methanogenesis within the system.^48^

Different GAC dosages (10, 20, and 30 g/L) resulted in significant differences in the volumetric production of biogas and methane in the reactors (Table 2 and Figure 3). However, the biogas compositions were similar (53–57% CH_4_). At the end of the semidry anaerobic digestion process of pretreated SM, the dosage of 10 g/L GAC (10PSM_GAC10_) resulted in a higher biogas production compared to dosages of 20 and 30 g/L GAC (10PSM_GAC20_ and 10PSM_GAC30_), producing on average 30% more biogas and 23% more methane. However, it is important to note in Figure 3C that, at the beginning of the experiment, 10PSM_GAC30_ has a cumulative methane yield slightly higher than that of 10PSM_GAC20_ and much higher when compared to that of 10PSM_GAC10_. Thus, while higher GAC concentrations accelerated initial methane production, the 10PSMGAC10 treatment surpassed others after day 55, reflecting better microbial adaptation. Over the 90 day period, the lower GAC concentration (10 g/L) proved more effective.

When analyzing the daily methane yield at 10% TS (Figure 3D), it is observed that 10PSM_GAC30_ achieved better results up to the 32nd day, with a maximum value of 8.0 mL of CH_4_/gVS on the 21st day, followed by 10PSM_GAC20_ with a maximum productivity of 5.4 mL of CH_4_/gVS on the 19th day. However, from the 39th day onward, 10PSM_GAC10_ started to present the highest daily methane yield, reaching a maximum value of 9.0 mL CH_4_/gVS on the 47th day, and continued with the best results until the end of the experiment.

However, in dry AD, the effect of the GAC dosage differed. When the TS content was increased from 10% to 15% using 10 g/L GAC, cumulative biogas and methane yields dropped by 80% and 90%, respectively. The methane content in the biogas was only 34%, which is below the typical range for anaerobic digestion (50–70%). In contrast, methane content was satisfactory at higher GAC concentrations (56–63% CH_4_ for 20 and 30 g/L GAC). Compared to semidry AD, reactors with 20 g/L GAC showed a 20% reduction in the final methane yield, while those with 30 g/L GAC showed no significant difference (Table 2). These findings align with the TOC results: a 30% TOC reduction was observed only at 30 g/L GAC (15PSM_GAC30_), while 15PSM_GAC20_ showed a minimal change between initial and final TOC levels. Notably, 10PSM_GAC10_ exhibited a 120% increase in the TOC by the end of the process, indicating that the methanogenic phase was ineffective.

This behavior is attributed to the limitations of dry AD, which include restricted substrate diffusion and adverse effects on microbial metabolism due to the reduced water content. At low GAC concentrations, the system lacks the resilience needed to counter these limitations. Consequently, mass transfer is hindered, impairing the assimilation of intermediate products and reducing the overall efficiency of methane production.^8,49^

Regarding the daily methane yield in dry AD (Figure 3F), the 15PSMGAC10 treatment reached its peak productivity on the first day; however, it was only 2.1 mL of CH_4_/gVS and remained consistently low throughout the experiment. In contrast, 15PSM_GAC20_ achieved its maximum yield on day 42 (4.7 mL of CH_4_/gVS) and maintained higher values than 15PSM_GAC30_ from day 55 onward. Notably, 15PSM_GAC30_ exhibited the highest peak yield of 6.4 mL of CH_4_/gVS, also on day 42. Analysis of the cumulative methane yield curve in dry AD (Figure 3E) confirmed the superior performance of the 30 g/L GAC condition, which can be attributed to its higher daily productivity between days 34 and 42.

As biogas production depends on the amount of organic matter in the reactor, the GAC concentration is also directly related to the organic load content. Previous studies have reported that increasing activated carbon concentration tends to result in a greater biogas volume when the load of organic matter increases.^50^

When raw swine manure was used, a variation in solid content was observed between the beginning and end of the experiment, indicating that the reactor with GAC and 10% total solids achieved greater reductions in both TS and VS, by 26% and 29%, respectively. This result suggests that GAC enhanced methanogenesis efficiency, as VS represents the main organic fraction responsible for methane production.^51^ In reactors without GAC, the TOC increased by 120% (10RSM) and 155% (15RSM). In contrast, reactors supplemented with GAC showed a decrease in soluble organic matter concentrations—65% for 10RSM_GAC20_ and 30% for 15RSM_GAC20_. These findings are consistent with the methane production trends observed in Figures 3C and 2E.

Reactors fed with raw or pretreated swine manure without the addition of conductive material produced between 56.4 and 68.8 mL/gVS of biogas but showed negligible methane production under semidry and dry AD conditions (3–8 mL/gVS). In contrast, the presence of GAC significantly improved performance, yielding 275.6 and 201.1 mL/gVS of biogas for total solids contents of 10% and 15%, respectively, and methane yields of 154 and 110 mL of CH_4_/gVS, respectively. Thus, increasing the TS content led to a ∼35% reduction in methane production.

With respect to daily methane production (Figures 3D and 2F), the highest value was observed for the 10RSM_GAC20_ condition, which reached 6.2 mL of CH_4_/gVS on day 15. In comparison, 15RSM_GAC20_ achieved a peak of 4.5 mL of CH_4_/gVS on day 19. Overall, daily methane productivity in 15RSM_GAC20_ was lower than that in 10RSM_GAC20_. This is attributed to the characteristics of swine manure at higher solids content (15%), where the substrate is more recalcitrant, resulting in slower hydrolysis and delayed conversion into methane during anaerobic digestion. As previously noted, significant methane production was only achieved in the presence of GAC.

Finally, it is also noted that in the present study, the thermoalkaline pretreatment did not promote a significant difference in biogas yield, methane yield, or methane composition between reactors with raw and pretreated manure, either in the absence or presence of 20 g/L GAC, under both evaluated conditions (10 and 15% TS). This suggests that while the pretreatment enhanced substrate solubilization, as evidenced by an approximately 38% increase in initial TOCs (see Table 1), it did not result in improved methane yield. This paradox can be attributed to the generation of inhibitory compounds during the pretreatment process—such as phenolic derivatives, furans (e.g., hydroxymethylfurfural), ammonia, and volatile fatty acids—which can negatively affect microbial syntrophy and methanogenic activity, particularly among sensitive archaeal species.^52–54^

When GAC was added at 20 g/L, methane production significantly increased in both raw (154 mL of CH_4_/gVS) and pretreated manure (155 mL of CH_4_/gVS), with no statistical difference between them. This result highlights the dual role of GAC: in raw manure, its primary function was to stimulate DIET, enhancing the electron exchange between syntrophic bacteria and methanogens and improving methane production in systems with low initial solubilization.^16^ In the pretreated manure, GAC’s effect was more likely due to its capacity to adsorb inhibitory compounds generated during the pretreatment,^55^ mitigating their toxicity and improving system stability.

Thus, although the initial substrate characteristics differed substantially between raw and pretreated manure, the action of GAC—either through conductivity or adsorptive detoxification—was sufficient to overcome the respective limitations, resulting in similar methane yields. This mechanistic insight reinforces the notion that GAC not only promotes DIET but also confers resilience to inhibitory pressures, making it a key additive in optimizing the anaerobic digestion performance across different substrate conditions.

Considering the positive effects of GAC on methane production, evaluating its economic feasibility in full-scale anaerobic digestion systems is crucial. Although a comprehensive economic assessment was beyond the scope of this study, the results show that the optimal GAC dosage varies with operational conditions, affecting cost-effectiveness. Lower concentrations (e.g., 10 g/L) performed better under semidry conditions, while higher concentrations (30 g/L) were necessary in dry systems. In practice, the economic feasibility of GAC implementation will depend on the market price, potential for regeneration or reuse, biogas valorization, and reactor configuration. Therefore, future studies should assess economic and life cycle aspects to support the large-scale application of GAC.

Moreover, the increased demand for GAC under dry conditions—where higher TS contents require up to three times more material—raises important concerns regarding material costs and sustainability.^15^ The possibility of reusing or regenerating GAC becomes a critical factor. Although regeneration techniques (e.g., thermal or chemical) have been shown to restore the adsorption capacity for multiple cycles, they often involve energy-intensive steps. They may require additional equipment, which can reduce the net environmental and economic benefits.^56^ Furthermore, scaling up GAC-based systems may face technical challenges related to reactor design, especially in high-solids processes where poor mixing and limited diffusion are common.^6^ In such cases, process adaptations, like internal mixing structures or fluidized-bed configurations, may be necessary to maintain contact between GAC particles and the microbial community, ensuring effective syntrophy and methane production. These findings underscore the importance of evaluating not only GAC performance but also its integration into operational strategies that consider cost-effectiveness, engineering feasibility, and long-term sustainability.

### 3.3 Ammoniacal Nitrogen Concentration and pH

The initial ammoniacal nitrogen and pH values are listed in Table 3. In general, throughout the experiment, there was an increase in ammonia concentration, which consequently led to an increase in pH. The ammonia level that may inhibit anaerobic digestion ranges from 1500 to 7000 mg/L in various substrates.^57^ Even the control reactors CT_S_ and CT_SG‑GAC_ were slightly above the inhibitory concentration for ammonia, demonstrating that the sludge used was rich in nitrogenous compounds. In dry AD, the ammonia nitrogen values were higher than in semidry AD, contributing to the lower methane production yields in reactors with 15% TS. Furthermore, reactors with activated carbon showed lower ammonia concentration at the end of the process, probably due to the adsorption capacity on the GAC surface.^58–60^

**Table 3:** Results of Soluble Ammonia Nitrogen and pH of the Reactors

reactors	soluble ammonia nitrogen (mg/L)		pH	
	initial	final	initial	final
CT_S_	288	1820	7.8	9.0
CT_SG_	291	714	7.9	8.9
CT_SG‑GAC_	286	1652	7.9	8.9
10RSM	633	3085	7.7	7.9
10RSM_GAC20_	535	2114	7.8	8.6
10PSM_N_	392	2235	7.6	8.0
10PSM	501	2352	7.7	8.2
10PSM_GAC10_	462	1997	7.5	8.5
10PSM_GAC20_	476	2091	7.7	8.6
10PSM_GAC30_	546	1526	7.5	8.3
15RSM	672	3304	7.7	8.2
15RSM_GAC20_	679	3033	8.0	9.0
15PSM	553	2968	7.7	8.6
15PSM_GAC10_	567	3211	7.9	8.5
15PSM_GAC20_	465	2417	8.0	9.0
15PSM_GAC30_	381	2641	7.8	9.0

Certainly, GAC also assisted in adsorbing other inhibitors besides ammonia throughout AD. Previous studies have shown that GAC effectively removes toxic substances, such as acids, *N*-heterocyclic compounds, phenols, heavy metals, and aromatic compounds, through its porous and rough surface, thereby aiding in the anaerobic digestion of high-strength organic substrates.^21,61^

Finally, as discussed in the previous section, reactors without conductive material or with nonconductive material exhibited lower methane yields. Although GAC can adsorb ammonia from the system, Table 3 shows that the final ammonia nitrogen concentrations under the GAC-supplemented conditions remained above the typical inhibition threshold. This supports the findings of Xiao et al.,^62^ who observed that the addition of GAC increases the system’s tolerance to ammonia inhibition. As a result, the risk of a decline in methane production due to excess ammonia is reduced.

### 3.4 Modeling the Kinetics of Methane Production

Table S4 presents all values of the reactor’s kinetic parameters and the error functions. Analyzing the CT_SG_ and CT_SG‑GAC_ control groups, the selected models presented a good fit to the experimental data, particularly the Transfer model. The addition of GAC in the glucose control resulted in a 13% increase in the first-order production rate of methane. However, there was no significant difference in the maximum methane production rate (μ_m_) and the lag phase (λ) reduction from 2.6 to 0 days. The presence of a conductive material favored the kinetics of methane production. The model that best described methane production in this study was Modified Gompertz, a model commonly used for describing AD of complex substrates. It provided the best fit to the experimental data, as indicated by high *R* ^2^ values and lower AIC values (Table 4).

**Table 4:** Parameters Estimated by the Modified Gompertz Model of Methane Production by Anaerobic Digestion^a^

parameters	reactors									
	CT_SG_	CT_SG‑GAC_	10RSM_GAC20_	10PSM_GAC10_	10PSM_GAC20_	10PSM_GAC30_	15RSM_GAC20_	15PSM_GAC10_	15PSM_GAC20_	15PSM_GAC30_
μ_m_ (mL/gVS·d)	1.93^a^	2.14^a^	3.36^b,d^	3.66^b,d^	3.59^b,d^	3.51^b,d^	2.08^a^	0.35^c^	3.00^d^	4.15^b^
λ (d)	2.21^a^	0.00^b^	6.50^c^	19.01^d^	10.43^e^	7.19^c^	10.62^e^	0.00^b^	31.98^f^	21.30^d^
*R* ^2^	0.993	0.889	0.993	0.997	0.988	0.979	0.990	0.920	0.991	0.999
AIC	119.13	251.32	178.66	165.94	220.95	250.62	161.63	32.79	169.70	93.63

a Note: Equal letters mean no significant difference (*p* < 0.05).

Among the kinetic coefficients estimated by the model, when using raw swine manure with 20 g/L of GAC, the μ_m_ was approximately 38% lower under dry conditions compared to semidry conditions, and the lag phase increased from 6.5 to 10.6 days. In the case of pretreated SM at the same GAC concentration, μ_m_ did not vary significantly with increased total solids content; however, the lag phase increased markedly from 10.4 to 32.0 days. These results indicate that increasing the total solids content reduced the maximum methane production rate for raw manure and significantly extended the lag phase for pretreated manure.

As previously discussed, this behavior reflects the challenges imposed by high total solids in AD, even when conductive materials, such as GAC, are used to enhance methane production efficiency. Under elevated TS conditions, the system faces greater mass transfer limitations and impaired microbial activity due to reduced water availability. Consequently, the diffusion of metabolites is hindered, affecting the assimilation of intermediate compounds and ultimately lowering both the rate and efficiency of methane production.^8,49^

However, when comparing raw and pretreated substrates, both supplemented with 20 g/L of GAC, no significant difference in the μ_m_ was observed under semidry AD conditions. Nonetheless, the lag phase increased from 6.5 to 10.4 days with pretreatment. Under dry conditions, pretreatment led to a 44% increase in μ_m_ but also extended the lag phase substantially—from 10.6 to 32.0 days.

Pretreatment can enhance methane production by breaking down complex organic matter into simpler, more bioavailable compounds for microbial uptake. However, it may also release inhibitory substances into the medium such as hydroxymethylfurfural, phenols, trace elements, sulfides, humic acids, and heavy metals. These compounds can hinder microbial adaptation, resulting in a prolonged lag phase.^52–54^

Concerning the GAC dosage study with pretreated swine manure (SM), no significant difference in the μ_m_ was observed under semidry AD conditions as the GAC concentration increased. However, the lag phase (λ) decreased from 19.0 to 10.4 and 7.2 days with GAC concentrations of 10, 20, and 30 g/L, respectively. Under dry conditions, the reactor with 10 g/L GAC exhibited the lowest μ_m_ and λ = 0, yet methane production occurred only during the initial days. This is likely due to early saturation of the GAC active sites, impairing both DIET and the adsorption of inhibitory compounds. Increasing the GAC concentration from 20 to 30 g/L resulted in a 38% increase in μ_m_ and a reduction in λ from 32.0 to 21.3 days.

Therefore, under higher TS conditions, improved kinetics are achieved with higher GAC concentrations, which enhance both methane production rates and microbial adaptation.

### 3.5 Microbial Community Analysis

Analysis of the microbial community was conducted at the end of the experiments for samples from reactors CT_S_, 10RSM, 10RSM_GAC20_, 10PSM, 10PSM_GAC10_, 10PSM_GAC30_, 15RSM, 15RSM_GAC20_, 15PSM, 15PSM_GAC10_, and 15PSM_GAC30_. The CT_S_, 10RSM, and 10PSM groups were also analyzed prior to the experiments. The alpha diversity was determined to report the species richness (estimated by Chao1) and diversity (determined by the Shannon index) (Table S5).

#### 3.5.1 Bacterial Communities

As shown in Figure 4A, at the bacterial phylum level at the beginning of the experiment, the compositions of CT_S_ and 10PSM were approximately the same, with the dominance of *Chloroflexi* (41.3 and 38.5%) and *Actinobacteriota* (15.7 and 14.2%). The hydrolytic-fermentative bacteria *Chloroflexi* is commonly found in anaerobic digestion systems processing livestock wastewater and sludge.^63^ *Actinobacteriota* participate in the acidogenesis process, producing acetate and hydrogen from simple organic matter.^64^ In the 10RSM sample, the dominant bacterial phylum was *Firmicutes* (55.8%), which are frequently found in animal manure and are associated with the hydrolysis and acidogenesis stages in AD.^65,66^

**Figure 4:**
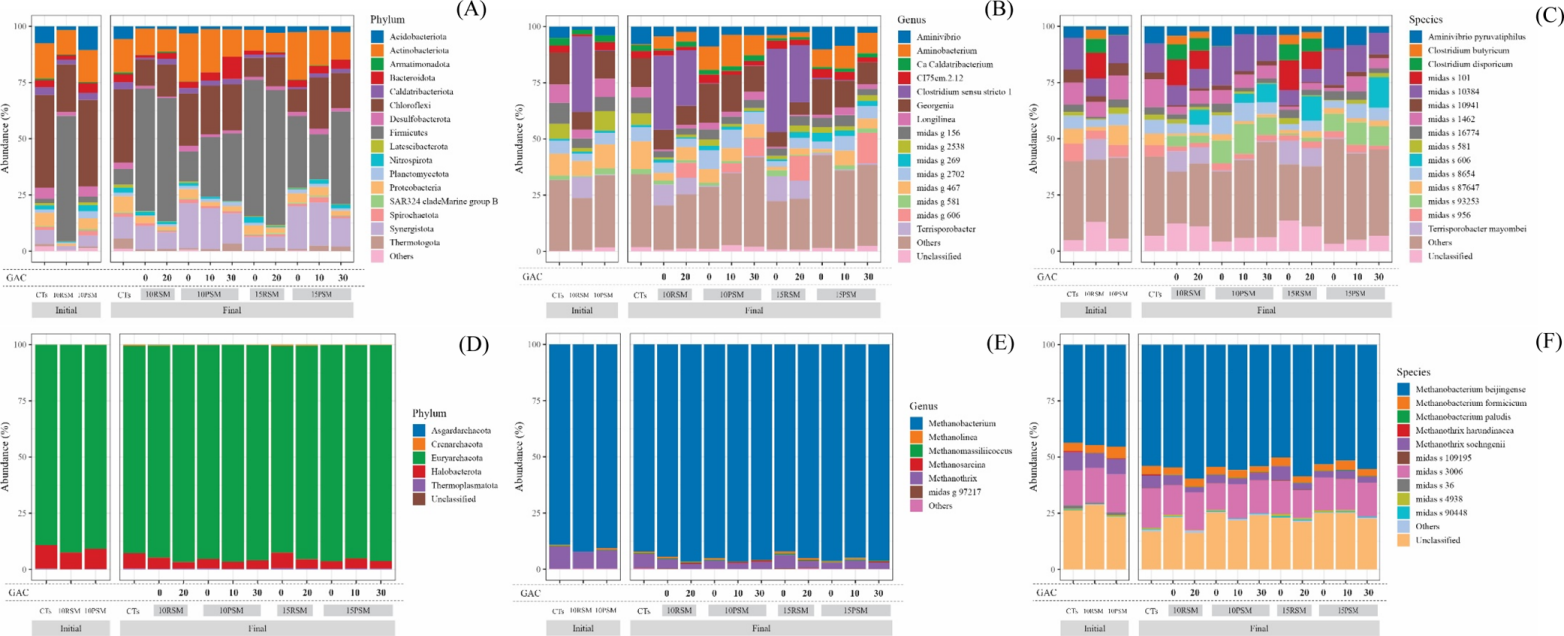
Taxonomic classification of bacteria (A–C) and archaea (D–F) at the phylum, genus, and species level.

At the end process, in samples containing raw manure, the genus *Clostridium sensu stricto 1* (phylum *Firmicutes*) predominates (Figure 4B), highlighting the *midas s 101* species (Figure 4C); while for pretreated manure, the genus *Georgenia* (phylum *Actinobacteriota*) predominates, highlighting the *midas s 10384* species. Although pretreatment generated differences in microbiota and increased the richness and diversity (Table S5), there was no significant difference in methane production, which may be a case of functional redundancy.

The dominance of *C. sensu stricto 1* in systems using raw swine manure suggests a pivotal role in the early stages of anaerobic digestion. This genus includes fermentative species that actively hydrolyze and acidify complex organics, producing acetate, hydrogen, and other volatile fatty acids, which are all critical precursors for methanogenesis. The consistent presence of*C. sensu stricto 1* in GAC-treated conditions may indicate a synergistic interaction with hydrogenotrophic methanogens, particularly under conditions favoring DIET. Although traditionally associated with indirect interspecies electron transfer (IET), some strains within *Clostridium* possess conductive pili, potentially linking them to DIET pathways when conductive materials are present.^63,67^

In contrast, the genus *Georgenia*, which exhibits greater abundance in reactors operated with pretreated manure, is not typically highlighted in the AD literature. However, studies have identified its capacity for oxidative stress tolerance and surface colonization, traits that may support biofilm development and microbial stability in high-soluble-organic environments. Additionally, *Georgenia* has demonstrated high tolerance to adverse environmental conditions and plays a key role in the hydrolysis of complex organic matter, thereby facilitating the degradation of toxic organic compounds.^68^ Notably, it is also considered a key protein-degrading bacterium, whose relative abundance tends to increase in anaerobic systems under high organic loads, especially when there is significant hydrolytic demand for proteins.^69,70^ Although no direct metabolic pathway involving DIET has been reported for *Georgenia*, its structural contribution to microbial consortia and its association with improved methane yields in GAC-supplemented systems suggest a supportive ecological function.

Analyzing the effect of adding GAC in the condition with raw manure at 10% TS (10RSM_GAC20_), there has been a discrete increase in Chao1 and Shannon indexes, and it is highlighted that the abundance of *Bacteroidota* and *Chloroflexi* increased from 1.2 and 11.8% to 2.8 and 14.3%. With 15% TS (15RSM_GAC20_), there was a reduction in these indexes, possibly because in a more difficult condition for microorganisms, due to the lower water content in the reactional medium, the presence of activated carbon selected bacteria more involved in the DIET process, reducing the richness and diversity of the microbial community. In addition, *Acidobacteriota* and *Chloroflexi* increased from 1.6 and 8.5% to 2.9 and 12.9%.

Therefore, the increase in the abundance of phyla *Bacteroidota*, *Chloroflexi*, and *Acidobacteriota* was linked to greater methane production when raw manure. Significant research has indicated that the enrichment of *Chloroflexi* and *Bacteroidota* is beneficial for macromolecular organic degradation and can strongly support methanogen metabolism (Y. Li et al., 2022; Zhuravleva et al., 2022).

It also stands out that the GAC addition promoted a significant increase in the abundance of *midas g 606*, which belongs to the *Firmicutes* phylum, the dominant phylum for conditions with raw manure. Using SILVA taxonomy^71^ (unpublished data), the same occurred for HN–HF0106 (*Firmicutes*). These cellulolytic bacteria can produce H_2_ and acetate using cellulose as their energy source and sole carbon.^63^ Therefore, it is an important microorganism to metabolize raw SM, which is rich in cellulose, due to the composition of the animal feed used in pig farming.

For the pretreated manure, in the semidry condition (10% TS), when adding GAC, there was an increase in the abundance of *Bacteroidota* (0 g/L GAC: 2.8%, 10 g/L GAC: 3.7%, 30 g/L GAC: 9.8%) and *Firmicutes* (0 g/L GAC: 13.5%, 10 g/L GAC: 26.6%, 30 g/L GAC: 30.0%). Furthermore, 10 g/L GAC caused practically no difference in the alpha diversity indexes. However, 30 g/L GAC reduced the diversity, suggesting a more specialized community. However, this makes the system more unstable, which may justify greater methane production with 10 g/L GAC. The dry condition showed different behavior concerning the abundance of *Firmicutes*: when adding 10 g/L GAC, it decreased from 31.9 to 19.9%, but with 30 g/L GAC (which showed the highest methane production on dry AD), it increased to 41.3%, and the Chao1 and Shannon indexes increased with the conductive material addition. Therefore, besides the phyla *Bacteroidota* and *Chloroflexi*, the *Firmicute* increase is also related to increased methane yield with pretreated manure.

#### 3.5.2 Archaea Communities

As shown in Figure 4D, *Euryarchaeota* was the most abundant archaea at the phylum level (89.2–96.6%), and *Halobacterota* was the second one (3.0–10.5%). These phyla are commonly found as microbial constituents in anaerobic systems.^69^ In general, at the end of the process, there was an increase in the abundance of *Euryarchaeota*. The phyla *Crenarchaeota* and *Thermoplasmatota* were virtually absent from the samples with abundances of 0.1–0.5%.

The hydrogenotrophic methanogen *Methanobacterium* (*Euryarchaeota* phylum) was the most abundant archaea at the genus level for all samples studied (Figure 4E), which suggests that hydrogenotrophic methanogenesis was the dominant pathway in these experiments. The second most abundant group was *Methanothrix* (*Halobacterota* phylum), a typical acetoclastic methanogen. It exhibits greater sensitivity to ammonia, VFAs, and other compounds than hydrogenotrophic methanogens.^62^ Previous studies have observed a significant positive correlation between *Georgenia* and *Methanothrix*, indicating that *Georgenia* not only provides substrates but also creates a favorable environment for methanogens, thereby promoting an improvement in the AD rate and increasing the overall stability of the reactor.^68^

In general, the addition of conductive material reduced the Chao1 (richness) and Shannon (diversity) indexes, suggesting a microbiota that is more specialized in methane production. No significant variations were observed in the relative abundances of the archaeal community. However, it stands out that the *Methanobacterium* genus was slightly more favored mainly when there was addition of GAC in the condition with raw manure. The most abundant species were *Methanobacterium beijingense* and *midas_s_3006* (Figure 4F). These results suggest that DIET can be established between *Methanobacterium* and *Methanothrix* archaea and *C. sensu stricto 1*, a bacteria genus favored by electron-conductive additives.^21^

Therefore, since there was no substantial variation in the archaeal community, it can be inferred that methane production was not inhibited by VFAs as no pH reduction was observed. As previously discussed, there was also no strong evidence of ammonia inhibition. These findings suggest that the primary limitation to methane production was the high TS content (10–15%), which hinders mass transfer processes and negatively affects microbial metabolism due to limited water availability in the medium. Thus, the results clearly demonstrate that the addition of GAC enhances the biomethanation process by promoting DIET.

## 4 Conclusions

Conductive materials can improve methane production exponentially in anaerobic digestion, even without pretreatment. In semidry and dry conditions, there was no significant difference in methane production yield when using raw or pretreated swine manure with the addition of 20 g/L GAC. However, there was an increase in the lag phase when using the pretreated substrate from 6.5 to 10.6 days and from 10.4 to 32.0 days at 10 and 15% TS, respectively. Therefore, when GAC addition is used, it is not necessary to pretreat this waste prior to the AD process. With raw manure, when the TS content was increased to 15%, even in the presence of GAC, the methane yield was reduced by 35%. For pretreated manure, the final methane production was higher at 10 g/L GAC for semidry condition (190 mL CH_4_/gVS). However, the process kinetics were more favorable for 20 and 30 g/L GAC (155 and 158 mL CH_4_/gVS), with a reduction in the lag phase from 19 to 10 and 7 days, respectively. For dry conditions, 10 g/L was not enough to promote satisfactory methane production (methane content in biogas of 25%–18 mL CH_4_/gVS), while 20 and 30 g/L GAC produced 127 and 157 mL CH_4_/gVS (biogas composition of 51 and 54% CH_4_), increasing the methane production rate from 0.4 to 3.0 and 4.2 mL/gVS·d. Therefore, the TS content influences the ideal GAC concentration in AD.

About microbiology, the increase in the abundance of phyla *Acidobacteriota*, *Bacteroidota*, *Chloroflexi*, and *Firmicutes* was linked to greater methane production when using raw manure. The dominant genus of bacteria in most reactors was *Georgenia*, and the main abundances of archaea were *Methanobacterium* (slightly more favored with GAC addition) and *Methanosaeta*. Therefore, the GAC addition increases methane production not only by favoring some important species in the AD process but also mainly by promoting DIET and adsorbing inhibitory compounds, thus improving methane-rich biogas production.

## Supplementary Material



## Data Availability

E-supplementary data of this work can be found in the online version of the paper.

## Notes

Detailed tables presenting the physicochemical characterization of fresh and pretreated swine manure and inoculum; the amounts of manure, sludge, and additive used in each experimental condition; XRF analysis of granular activated carbon; parameters estimated by kinetic modeling of methane production via anaerobic digestion; and the alpha-diversity indices of each analyzed sample (PDF)

## Abbreviations

10PSM: pretreated swine manure + sludge (10% TS)
10PSM_GAC10_: pretreated swine manure + sludge +10 g GAC/L (10% TS)
10PSM_GAC20_: pretreated swine manure + sludge +20 g GAC/L (10% TS)
10PSM_GAC30_: pretreated swine manure + sludge +30 g GAC/L (10% TS)
10PSM_N_: pretreated swine manure + sludge +20 g/L nylon (10% TS)
10RSM: raw swine manure + sludge (10% TS)
10RSM_GAC20_: raw swine manure + sludge +20 g GAC/L (10% TS)
15PSM: pretreated swine manure + sludge (15% TS)
15PSM_GAC10_: pretreated swine manure + sludge +10 g GAC/L (15% TS)
15PSM_GAC20_: pretreated swine manure + sludge +20 g GAC/L (15% TS)
15PSM_GAC30_: pretreated swine manure + sludge +30 g GAC/L (15% TS)
15RSM: raw swine manure + sludge (15% TS)
15RSM_GAC20_: raw swine manure + sludge +20 g GAC/L (15% TS)
AD: anaerobic digestion
AIC: akaike information criterion
ANOVA: analysis of variance
ASVs: amplicon sequencing variants
COD_S_: chemical oxygen demand of soluble fraction
CT_S_: Sludge (10% TS)
CT_SG_: sludge + glucose (10% TS)
CT_SG‑GAC_: sludge + glucose +20 g GAC/L (10% TS)
DIET: direct interspecies electron transfer
GAC: granular activated carbon
NCBI: National Center for Biotechnology Information
SM: Swine Manure
TAN_S_: total ammonia nitrogen of soluble fraction
TOC_S_: total organic carbon of soluble fraction
TS: total solids
VS: volatile solids
