# Supplementary material for: Influence of Granular Activated Carbon Addition on Methane Production in Dry and Semidry Anaerobic Digestion of Swine Manure
Source: ACS Omega. 2025 Jul 7;10(27):29386–99. doi: 10.1021/acsomega.5c02516 (PMC12268741; doi:10.1021/acsomega.5c02516)
Supplement: Supplementary file 1 [file ao5c02516_si_001.pdf]

Supplementary material

Influence of granular activated carbon addition on methane production in dry and semi-dry anaerobic digestion of swine manure

Amanda de Sousa e Silva[a], Amanda Lima Moraes dos Santos[a], Isabele Clara Cavalcante Malveira[a], Bianca Holanda Albano Girão[a] and André Bezerra dos Santos[a,*]

[a] Department of Hydraulic and Environmental Engineering, Federal University of Ceará, Fortaleza, Ceará, Brazil.

[*]Corresponding author: Department of Hydraulic and Environmental Engineering. Campus do Pici, Bloco 713. Pici. CEP: 60455-900. Fortaleza – Ceará – Brazil. Phone/Fax: +55 85 3366-9490. E-mail: andre23@ufc.br

Table S1. Physicochemical characterization of fresh and pretreated swine manure and inoculum.

Parameter	Raw swine manure	Pretreated swine manure	Inoculum
**Total solids - TS (%)**	23.5	14.0	12.4
**Volatile solids - VS (%)**	18.9	8.4	10.2
**VS/TS (%)**	80.5	59.9	82.5
**pH**	5.7	7.0	6.8
**COD_S^* (g/L)**	65.2	39.3	3.2
**TAN_S^* (mg/L)**	446.9	351.7	288.4

Legend: COD_S - soluble Chemical Oxygen Demand; TAN_S - soluble ammoniacal nitrogen.

Table S2. Amounts of manure, sludge and additive used in each condition.

Reactors	Raw manure (g/L)	Pretreated manure (g/L)	Sludge (g/L)	GAC (g/L)
**CT_S**	-	-	763	-
**CT_{SG}**	-	-	763	-
**$CT_{SG\text{-}GAC}$**	-	-	763	20
**10RSM**	212	-	382	-
**$10RSM_{GAC20}$**	212	-	382	20
**10PSM**	-	409	326	-
**$10PSM_N$**	-	409	326	*
**$10PSM_{GAC10}$**	-	409	326	10
**$10PSM_{GAC20}$**	-	409	326	20
**$10PSM_{GAC30}$**	-	409	326	30
**15RSM**	323	-	597	-
**$15RSM_{GAC20}$**	323	-	597	20
**15PSM**	-	461	537	-
**$15PSM_{GAC10}$**	-	461	537	10
**$15PSM_{GAC20}$**	-	461	537	20
**$15PSM_{GAC30}$**	-	461	537	30

Legend: * 20g/L of nylon; CT_S – sludge; CT_{SG} – sludge + glucose; $CT_{SG\text{-}GAC}$ – sludge + glucose + 20 g GAC/L; 10RSM – raw swine manure + sludge (10% TS); $10RSM_{GAC20}$ – raw swine manure + sludge + 20 g GAC/L (10% TS); 10PSM – pretreated swine manure + sludge (10% TS); $10PSM_N$ – pretreated swine manure + sludge + 20 g/L nylon (10% TS); $10PSM_{GAC10}$ – pretreated swine manure + sludge + 10 g GAC/L (10% TS); $10PSM_{GAC20}$ – pretreated swine manure + sludge + 20 g GAC/L (10% TS); $10PSM_{GAC30}$ – pretreated swine manure + sludge + 30 g GAC/L (10% TS); 15RSM – raw swine manure + sludge (15% TS); $15RSM_{GAC20}$ – raw swine manure + sludge + 20 g GAC/L (15% TS); 15PSM – pretreated swine manure + sludge (15% TS); $15PSM_{GAC10}$ – pretreated swine manure + sludge + 10 g GAC/L (15% TS); $15PSM_{GAC20}$ – pretreated swine manure + sludge + 20 g GAC/L (15% TS); $15PSM_{GAC30}$ – pretreated swine manure + sludge + 30 g GAC/L (15% TS).

Table S3. XRF analysis of granular activated carbon.

**Elements**	**Al_2O_3**	**SiO_2**	**SO_3**	**Cl**	**K_2O**	**CaO**	**TiO_2**	**Fe_2O_3**	**SeO_2**	**SrO**
**(%)**	7.64	35.66	21.41	1.49	1.16	11.83	12.34	8.04	0.22	0.18

Table S4. Parameters estimated by kinetic modeling of methane production by anaerobic digestion.

Model	Parameters	Reactors									
		CT_{SG}	CT_{SG-GAC}	$10RSM_{GAC20}$	$10PSM_{GAC10}$	$10PSM_{GAC20}$	$10PSM_{GAC30}$	$15RSM_{GAC20}$	$15PSM_{GAC10}$	$15PSM_{GAC20}$	$15PSM_{GAC30}$
**First Order**	**k(1/d)**	0.029[a]	0.036[b]	0,027[a]	0.017[c]	0.024[a]	0.026[a]	0,021[c]	0.031[a]	0.013[c]	0.019[c]
	**R^2**	0.966	0.969	0,940	0.841	0.908	0.930	0,905	0.968	0.733	0.815
	**AIC**	205,044	176,200	300,934	391,536	336,795	318,301	285,149	-21.530	368,331	375,845
**Second Order**	**k'' (L/g.d)**	$5.3\cdot10^{-4}$[a]	$7.3\cdot10^{-4}$[a]	$2.9\cdot10^{-4}$[c]	$1.2\cdot10^{-4}$[c]	$2.5\cdot10^{-4}$[c]	$2.7\cdot10^{-4}$[c]	$2.9\cdot10^{-4}$[c]	$3.3\cdot10^{-3}$[d]	$1.3\cdot10^{-4}$[c]	$1.8\cdot10^{-4}$[c]
	**R^2**	0.854	0.955	0,820	0.730	0.793	0.822	0,788	0.929	0.630	0.692
	**AIC**	289,399	196,713	365,044	422,937	384,699	373,684	331,077	23,606	386,847	404,998
**Monomolecular**	**k(1/d)**	0.032[a]	0.036[a]	0,032[a]	0.021[b]	0.030[a]	0.031[a]	0,026[a]	0.031[a]	0.018[b]	0.025[a,b]
	**λ (d)**	2.617[a]	0.000[b]	4,698[c]	9.181[d]	6.243[c]	5.217[c]	7,067[c]	0.000[b]	11.906[e]	9.091[d]
	**R^2**	0.975	0.969	0,968	0.906	0.953	0.965	0,952	0.968	0.812	0.882
	**AIC**	188,924	178,200	266,614	362,751	299,130	280,177	248,228	-19,530	350,277	352,172
**Logistics**	**μ_m (mL/gVS.d)**	1.846[a]	1,984[a]	3,253[b]	3.664[b]	3.616[b]	3.458[b]	2,007[a]	0.322[c]	2,940[b]	4,140[b]
	**λ (d)**	2.621[a]	0.000[a]	7,254[b]	21.154[c]	11.791[b]	8.131[b]	11,676[b]	0.000[a]	33.519[d]	22.606[c]
	**R^2**	0.980	0.854	0,978	0.989	0.968	0.954	0,971	0.908	0.994	0.993
	**AIC**	175,922	267,567	246,207	233,671	277,211	296,385	219,679	40,820	150,862	192,893
**Modified Gompertz**	**μ_m (mL/gVS.d)**	1.929[a]	2.142[a]	3.362[b,d]	3.655[b,d]	3.591[b,d]	3.506[b,d]	2.084[a]	0.347[c]	2.998[d]	4.145[b]
	**λ (d)**	2.211[a]	0.000[b]	6.503[c]	19.007[d]	10.425[e]	7.193[c]	10.617[e]	0.000[b]	31.983[f]	21.301[d]
	**R^2**	0.993	0.889	0,993	0.997	0.988	0.979	0,990	0.920	0.991	0.999
	**AIC**	119,131	251,324	178,661	165,941	220,946	250,618	161,628	32,791	169,704	93,628
**Transfer**	**μ_m (mL/gVS.d)**	3.042[a]	3.368[a]	4,846[b]	3.941[a,b]	4.566[b]	4,890[b]	2,809[a]	0.551[c]	2,260[a]	3,860[a,b]
	**λ (d)**	2.594[a]	0.000[b]	4,682[c]	9.145[d]	6.227[c]	5,200[c]	7,036[c]	0.000[b]	11.878[e]	9.076[d]
	**R^2**	0.975	0.968	0,968	0.906	0.953	0.964	0,953	0.968	0.810	0.881
	**AIC**	187,393	177,446	265,443	359,827	297,986	278,981	245,695	-21.423	347,595	351,157

Legend: CT_{SG} – sludge + glucose; CT_{SG-GAC} – sludge + glucose + 20 g GAC/L; $10RSM_{GAC20}$ – raw swine manure + sludge + 20 g GAC/L (10% TS); $10PSM_{GAC10}$ – pretreated swine manure + sludge + 10 g GAC/L (10% TS); $10PSM_{GAC20}$ – pretreated swine manure + sludge + 20 g GAC/L (10% TS); $10PSM_{GAC30}$ – pretreated swine manure + sludge + 30 g GAC/L (10% TS); $15RSM_{GAC20}$ – raw swine manure + sludge + 20 g GAC/L (15% TS); $15PSM_{GAC10}$ – pretreated swine manure + sludge + 10 g GAC/L (15% TS); $15PSM_{GAC20}$ – pretreated swine manure + sludge + 20 g GAC/L (15% TS); $15PSM_{GAC30}$ – pretreated swine manure + sludge + 30 g GAC/L (15% TS); k: methane production

rate first-order constant (1/d). K": methane production rate second-order constant (1/d). λ: time of the lag phase (d). μ_m: maximum rate of methane production (mL CH_4/gVS.d). R^2: coefficient of determination; AIC: Akaike Information Criterion. Equal letters mean no significant difference ($p < 0.05$).

Table S5. The alpha-diversity indices of each sample.

Reactor	Condition	Chao1		Shannon	
		Bacterial	Archaea	Bacterial	Archaea
CT_S	Initial	650.10	366.20	6.24	5.57
10RSM		736.75	358.14	6.37	5.53
10PSM		834.37	419.20	6.46	5.67
CT_S	Final	911.06	416.00	6.51	5.52
10RSM		902.09	400.20	6.51	5.43
$10RSM_{GAC20}$		933.53	374.50	6.58	5.37
10PSM		804.00	431.00	6.38	5.42
$10PSM_{GAC10}$		823.08	408.00	6.37	5.40
$10PSM_{GAC30}$		576.50	259.00	6.02	5.28
15RSM		793.11	386.43	6.35	5.49
$15RSM_{GAC20}$		686.05	278.00	6.30	5.23
15PSM		461.15	343.33	5.81	5.32
$15PSM_{GAC10}$		674.05	402.00	6.27	5.44
$15PSM_{GAC30}$		490.15	305.00	5.91	5.31

Legend: CT_S – sludge; 10RSM – raw swine manure + sludge (10% TS); $10RSM_{GAC20}$ – raw swine manure + sludge + 20 g GAC/L (10% TS); 10PSM – pretreated swine manure + sludge (10% TS); $10PSM_N$ – pretreated swine manure + sludge + 20 g/L nylon (10% TS); $10PSM_{GAC10}$ – pretreated swine manure + sludge + 10 g GAC/L (10% TS); $10PSM_{GAC30}$ – pretreated swine manure + sludge + 30 g GAC/L (10% TS); 15RSM – raw swine manure + sludge (15% TS); $15RSM_{GAC20}$ – raw swine manure + sludge + 20 g GAC/L (15% TS); 15PSM – pretreated swine manure + sludge (15% TS); $15PSM_{GAC10}$ – pretreated swine manure + sludge + 10 g GAC/L (15% TS); $15PSM_{GAC30}$ – pretreated swine manure + sludge + 30 g GAC/L (15% TS).
